# Bénédict Augustin Morel (1809-1873): The Originator of the Degenerative Theory of the Heredity of Mental Disorders and the Pioneer of the Biological Approach to Psychiatry

**DOI:** 10.7759/cureus.69523

**Published:** 2024-09-16

**Authors:** Eleni Rosiou, Markos Sgantzos, Grigoris Abatzoglou, Niki Papavramidou

**Affiliations:** 1 Department of History of Medicine, School of Medicine, Aristotle University of Thessaloniki, Thessaloniki, GRC; 2 Department of Morphology, School of Medicine, University of Thessaly, Larissa, GRC; 3 Department of Child and Adolescent Psychiatry, School of Medicine, Aristotle University of Thessaloniki, Thessaloniki, GRC

**Keywords:** alcoholism, criminality, degeneration, eugenics, heredity, historical vignette, mental disorders, psychiatry

## Abstract

The concept of progressive hereditary degeneration, which significantly influenced medical, particularly psychiatric and in turn social thought of the second half of the 19th century, was articulated by Bénédict Augustin Morel. The distinguished French psychiatrist developed the theory of degeneration and created the nosological framework of the heredity of mental illness in order to explain the more frequent psychoses and nervous disorders. In the absence of patho-anatomical findings, Morel attributed these phenomena to hereditary causes. His theory was the first attempt to interpret insanity, mental disorders, and criminality, across generations, and formed the basis for the further development of psychiatry. It had a notable influence on many scientific disciplines of the time, such as criminology, anthropology, biology, and general pathology. It would later result in the emergence of eugenics, which raised several moral issues and would ultimately be used in many ways to justify segregation. Morel was mainly influenced by the monogenetic degenerative theory and believed that social progress could be achieved by the coupling of psychiatry, a social medicine, with philosophy and Christianity.

## Introduction and background

**Figure 1:**
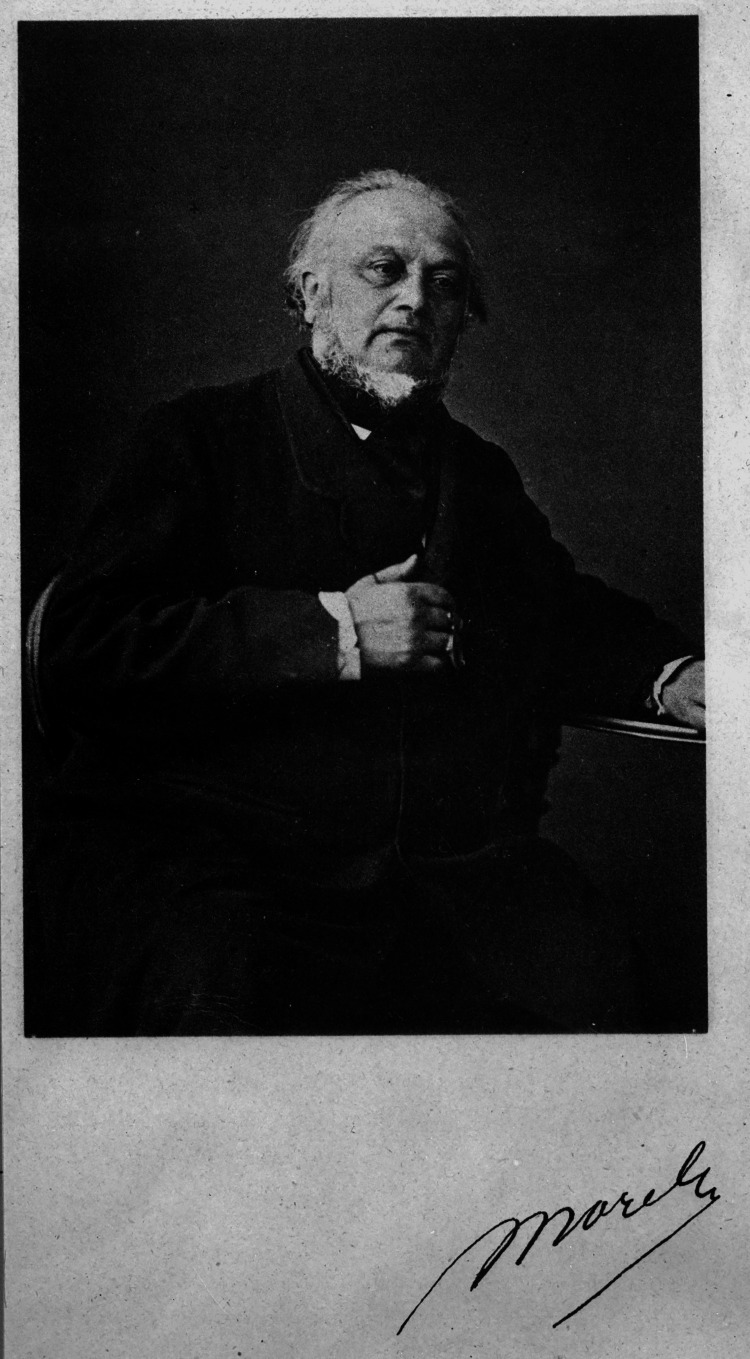
Morel, Bénédict Augustin (1809-1873). Image credits: Obtained from Bibliothèques d'Université Paris Cité. Permission: Image is under the Creative Commons Public Domain.

The main purpose of the article is to present the work of Bénédict Augustin Morel (Figure 1), the psychiatrist whose theory dominated Western psychiatry for many decades of the 19th and 20th centuries.

In the 1830s and 1840s in France, medicine came under fire for its failure to deal with the cholera epidemic that broke out in 1832. It therefore had to redefine itself after the Revolution of 1848 and develop a strategy to address the new challenges of industrialisation. The mortality rates, the social relations, and the dissatisfaction felt by many as a result of the economic crisis and the political conflicts of those years had to be studied and solutions had to be proposed. Psychiatry, as social medicine, would be the answer to all these problems together with philosophy, Christianity, and anthropology, with the common goal of physical, spiritual, and mental improvement, especially of the workers, and the preservation of order [1].

In the middle of the 19th century, as Morel mentions in his ‘’Treatise of Physical, Mental and Moral Degenerations of the Human Species and the Causes Which Produce the Morbid Varieties’’, a great increase was found in the admissions of mental patients to the asylum. A further increase was noticed in the cases of insanity, general paralysis, epilepsy, and general physical and mental debility, as well as neuroses, such as hysteria and hypochondria, accompanied by suicidal tendencies, which mainly concerned the classes of workers and rural dwellers. Morel, coming into contact with inmates of many European mental hospitals, observed their actions, the nature of their delirium, the shape of their heads, and dealing also with cases outside asylums, studied ‘everything abnormal either in outward appearance or in matters of morality’ [2].

**Figure 2:**
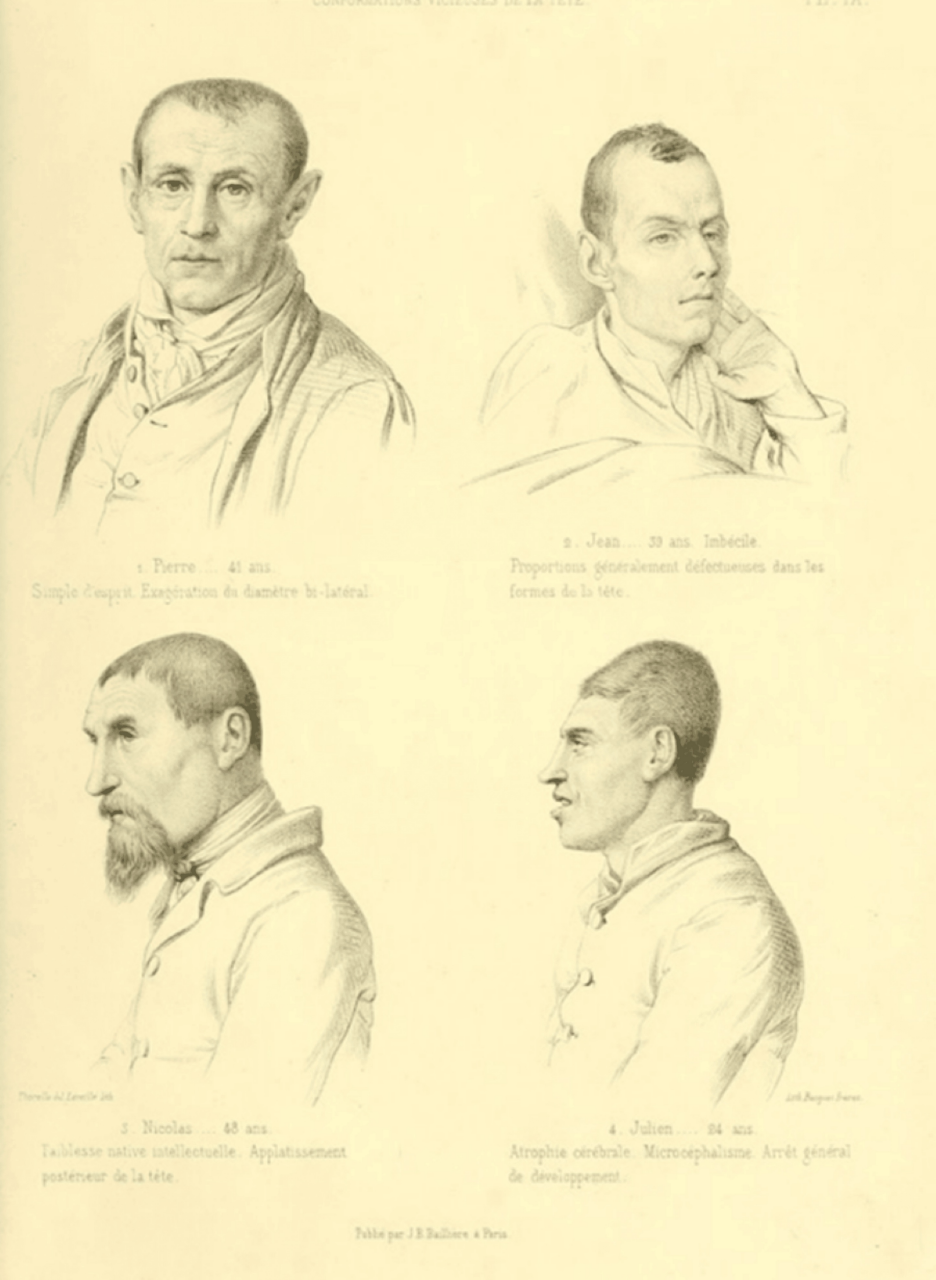
Siblings from parents of low intelligence, with abnormal head measurements. The existence of ear deformity and narrow chest width are some "signs" of their deviation from the normal human type. Image credits: Obtained from Bibliothèques d'Université Paris Cité. Permission: Image is under the Creative Commons Public Domain.

Morel’s goal was to classify all mental illnesses based on their cause and to create a nosological framework [3]. In these situations, as he characteristically stated, there is a "typical seal", some "spots", considered by Morel as the external signs of degeneration, such as the asymmetries of the head, ear deformities (Figure 2), height, genital growth retardation, various chronic diseases and hereditary malformations, strabismus, clubfoot, rickets, adding even the improper use of language, some ideas and instincts, and the incomplete intellectual development [2].

Morel was brought up Christian, admired the German school of psychology, as he believed it combined medicine and philosophy and was inspired by his friend, psychiatrist Philippe Buchez (1796-1865), to engage in religion and philosophy, wishing to combine Catholicism with social progress [1]. Clearly influenced by anthropological methods of classifying races, he argued that the shape of the head is an absolute function of the development or otherwise of mental functions [2]. Morel was also influenced by the work of Georges-Louis Leclerc, known as Comte de Buffon (1707-1788), who theorized that all animal and plant species evolved from primitive life forms and reacted to climatic and environmental changes by changing their characteristics, a process that the French mathematician and naturalist described as "degeneration" [4]. In the introduction to his first study, Morel states that he was influenced by Gall's theory of phrenology, which argued that mental and emotional functions can be detected by visual inspection of the skull, and by Flourent's anthropological studies [2], which dealt with the ideas of Cuvier (1769-1832), who treated racial inequality as an actual and undeniable outgrowth of natural organization, considering that there are internal causes that seem to halt the progress of certain races [5].

Morel’s theory was the first attempt to interpret insanity, mental disorders, and criminality, across generations. Mental illness was, for him, the result of pathological phenomena and could be inherited, leading to the degeneration and eventual extinction of the generation. Morel would describe the term "degeneration", a "morbid deviation of the species", including the concept of a pathological transformation that occurs in the perfect man, as God had created him at the beginning of time, in his primitive archetype [2].

## Review

Morel’s life and career

Bénédict Augustin Morel was born in 1809 in Vienna, Austria, to French parents. He was raised by the Luxembourger Abbé Dupont. He graduated from medical school in 1839, and two years later he was hired as the renowned Jean-Pierre Falret's secretary at the Salpêtrière hospital in Paris, France. After being appointed director of the mental hospital at Saint-Yon in 1856, he worked there until his death from diabetes in 1873 [6].

Due to the aforementioned conditions that prevailed in 19th century France, Morel called for the creation of the Higher Council of Public Health within the framework of an ambitious national plan as well as "free medicine" for the proletariat, with the first President of France (1848-1852) and then Emperor (1852-1870), Napoléon III (1808-1873) playing a very important role in this direction. Napoléon III showed a particular interest in Morel's work [1].

Morel’s Treatise of Degeneration was regarded at the time as highly philosophical and of moral value, and for this reason, its author was awarded a gold medal by the Institute of France, accompanied by a prize of 2,500 francs, in 1856. He was crowned a member of the Faculty of Sciences of the Academy of Medicine, served as vice-president of Rouen’s Society of Medicine, and was knighted in the Legion of Honor in 1864 [1].

Causes of degeneration

Morel's basic principle is that the degradation of the human species in addition to endogenous factors is also the result of exogenous ones but also social institutions or some occasional, case-by-case effects, with violations of the moral law and the lack of spiritual culture being factors that destabilize normal development [2].

**Figure 3:**
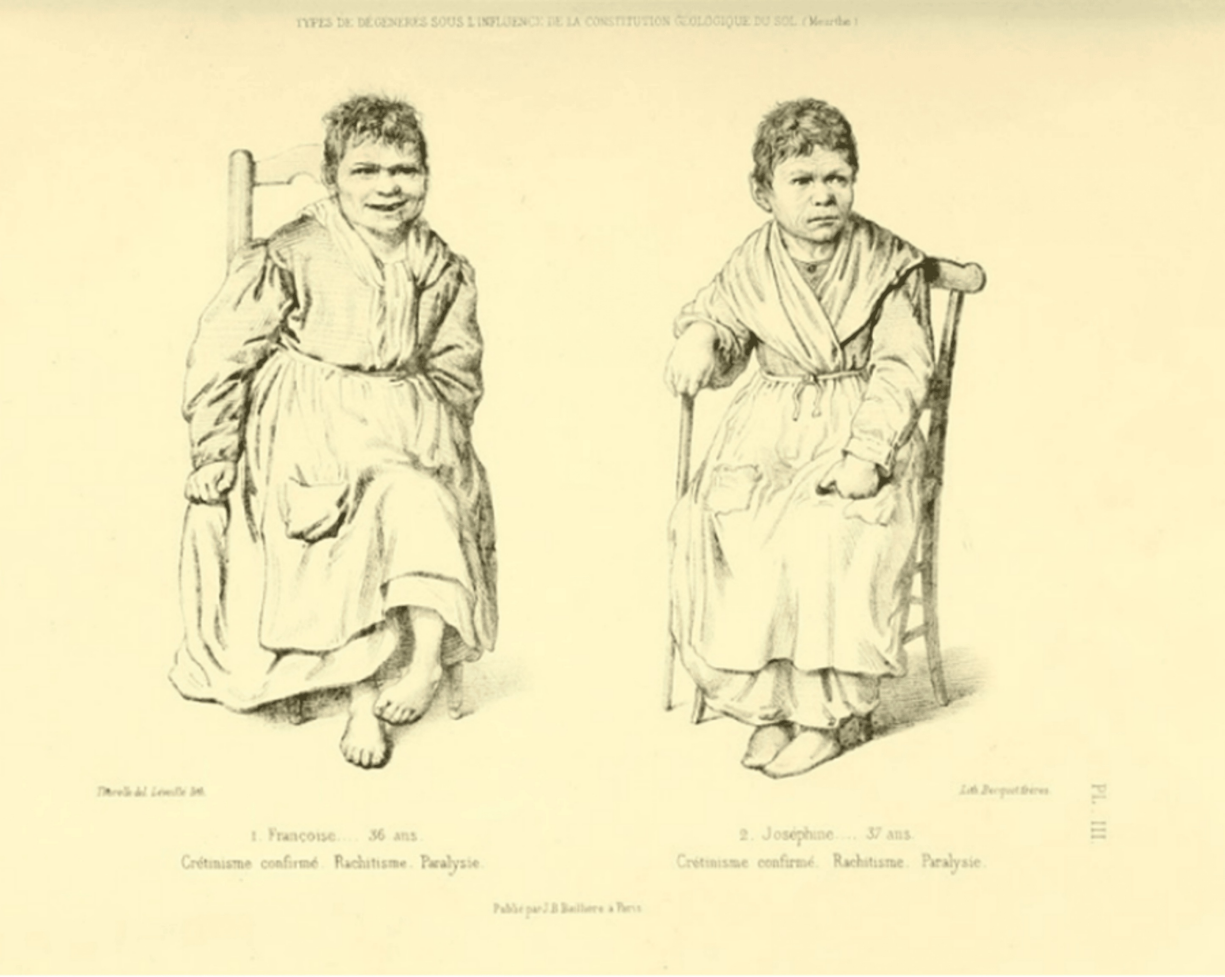
Cases of two degenerate cretinous women due to the condition of the soil - loamy and calcareous - of the Meurthe area where they lived. They have common features in their physiognomy, mental state, rachitic deformity, and reproductive incapacity. Image credits: Obtained from Bibliothèques d'Université Paris Cité. Permission: Image is under the Creative Commons Public Domain.

Morel, in the first part of his book, mentions intoxication as one of the causes of degeneration. Those who live in areas affected by malaria are likely to be led to hereditary cachexia, but there are also cases where degeneration can occur due to depravity, violation of hygiene rules, and the use of alcohol and drugs, such as opium. These factors cause serious perversions in the functions of the nervous system. Other causes mentioned in this section are the condition of the soil, famines, epidemics, and the quality of air and food (Figure 3). It is noteworthy that Morel claims that these conditions should be of long duration and epidemic form and are also a cause of degeneration not only of individuals but also of races. In regard to the existence of "varieties", borrowing here the anthropological term, that is, different races of the human species, Morel argues that these are due to natural changes, while at the same time, there are also degenerate human races. In the first case, if they arose from a disease, it simply did not have the power to prevent the continuation of the species [2].

Dangerous and unhealthy occupations, such as in factories and mines, as well as living in unhealthy or crowded places, weaken the organism and lead it to degeneration. If we add the factors of insufficient education, sexually transmitted diseases, or insufficient food, we will be led to the conclusion that the degeneration is a consequence of the social level and concerns the penurious class [2].

A large chapter of this theory is devoted to chronic alcoholism, which constitutes for Morel one of the main causes of the degradation of the species and relates this harmful habit to the phenomenon of heredity, which, as he characteristically notes, "is not an isolated event" but includes possible tendencies several generations before. The manifestations of degeneration in which these hereditary predispositions are found concern not only the external features such as small size or distorted skull shape, weak temperament, specific deformities, abnormalities in the structure of organs, or inability to reproduce but also the deviations found in the field of mental functions and emotions [2].

**Figure 4:**
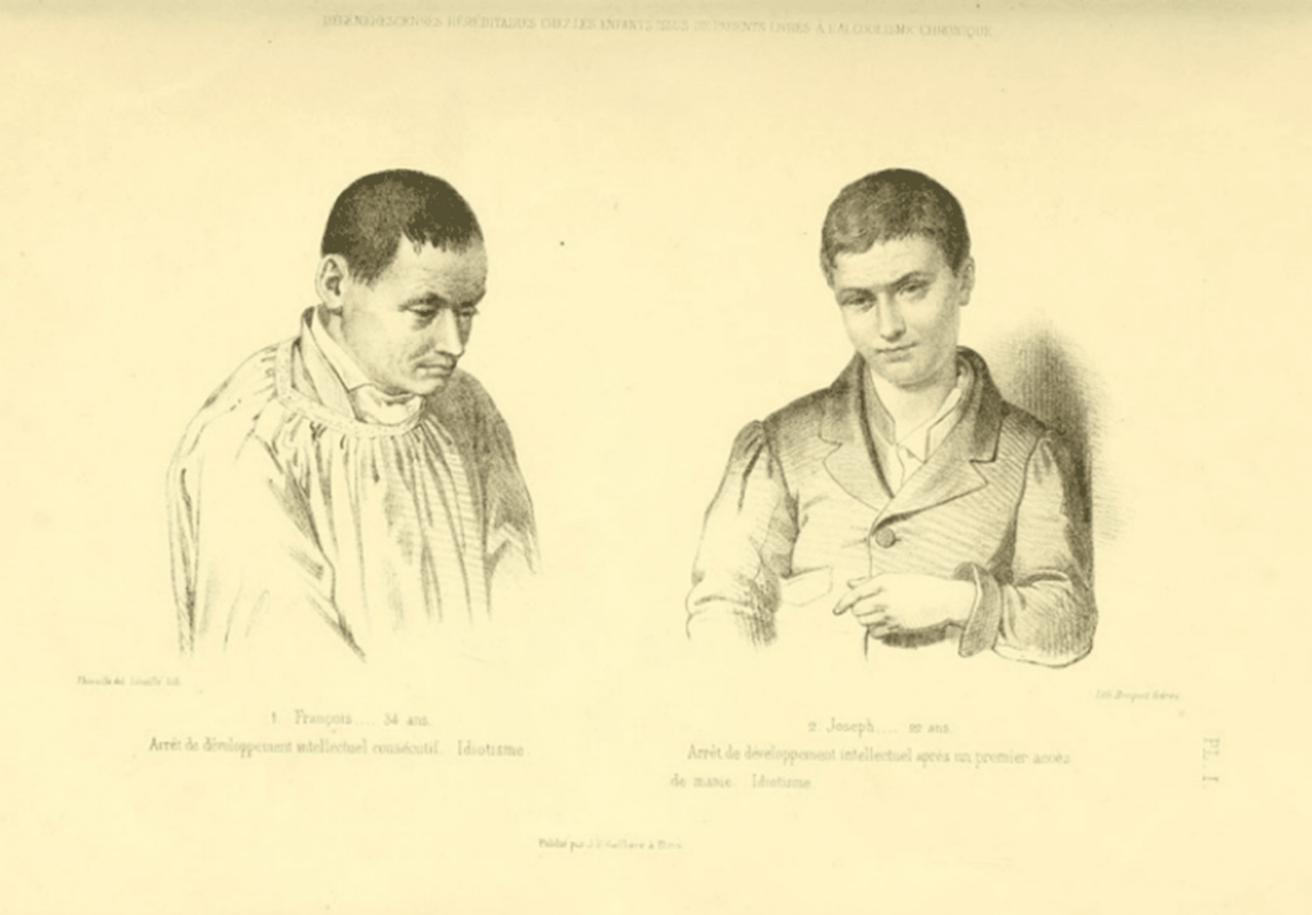
Offspring of alcoholics who exhibited growth failure and lack of reproductive capacity. Image credits: Obtained from Bibliothèques d'Université Paris Cité. Permission: Image is under the Creative Commons Public Domain.

Morel's conclusions, after observing cases of chronic alcoholism, are listed as follows: In the first generation of the offspring of the alcoholic subject, immorality, depravity, and excessive consumption of alcohol can be observed, as well as brutalization when deprived of alcohol. In the second, the tendency to alcoholism can be inherited and manic episodes and general paralysis can occur. In the third, sobriety, hypochondriac tendencies, melancholy, and suicidal tendencies can be observed. In the fourth generation, mental development may be lacking, the first manifestations of mania may arise at the age of 16, and idiocy and stupidity may appear, with the result that the alcoholic subject leads their generation to extinction [2] (Figure 4).

Morel also presents as other causes of intoxication and consequently degeneration, the use of hemp, hashish, and tobacco, while he refers extensively to the effect of lead, copper, phosphorus, and mercury on those who work in industrial units where these metals are used, as well as arsenic [2].

Organic lesions, which are the result of intoxication or other degenerative causes, appear either in an acute or chronic form. In the acute form, the injurious action may be so rapid that even the most meticulous autopsy may sometimes fail to reveal any organic alteration. In the chronic form, intoxication from alcohol, hashish, or opium, for example, can act progressively and cause irreversible symptoms. After all, the most active causes of degeneration are those that directly attack the brain and create instant insanity for the one who makes use of these toxic substances. The hereditary transmission carried out in such a way makes the nervous system capable of periodically reproducing such phenomena, which creates a new cerebral property with the result that the pathological changes are successive and create a "vicious circle". Disturbance of the mental state is also brought about by numbness, cramps, and damage to mobility and sensitivity, and these also initiate, according to Morel, a series of pathological phenomena. Convulsions, paralysis, and dementia are conditions that mark the progression of the disease, and the organic lesions that the autopsy can find at this stage are the result of the changes [2].

The above-mentioned periodicity of morbid phenomena results in the development of neuropathies, as seen in hysteria, epilepsy, intermittent fever, or in the final stages of some diseases. It is noteworthy that hysterics, epileptics, or hypochondriacs show common characteristics, the same habits, the same instincts, and even the same expression in the physiognomy. For the French psychiatrist, albinism, elephantiasis, and goiter are also degenerative aberrations [2].

The physical breakdown experienced by alcoholics and opium smokers has no specific external "stamp" or identical lesions. Deviation from the normal type of human is revealed by both internal and external signs in succeeding generations, such as incapacity, the manifestation of the worst tendencies, and the limitation of mental capacities to such an extent that the individual cannot fulfill their duties. In other words, these pathological differences concern the physical as well as the mental and psychic levels and are of course of major importance [2].

The incarcerated and the role of medicine

As Morel mentions, those who are locked up in mental asylums are a public danger because of their impulses and delusional acts. Those who experience these pathological conditions should not reproduce, as their offspring would have characteristics of morbid deviance. That is why medical intervention would be very useful in such cases [2].

About crime

Having made efforts to differentiate insanity from crime, he found that the majority of those suspected of criminal acts were in a phase of "incubation of insanity", while in the rest the active causes were immorality, poverty, drunkenness, situations which, according to Morel, created mental confusion [2].

As for the increase in juvenile delinquency among minors under the age of fifteen, a very alarming and serious problem, the French psychiatrist reports that he found many cases of hereditary criminality among young prisoners, whose stunted growth and defective head formation clearly revealed the cause. "I was overcome with a deep feeling of sorrow when I thought that beings who deviated from the normal type of man were destined one day to spread degeneracy", comments Morel [2].

Ways to reform the human species

In addition to the exhortations scattered throughout his treatise to improve living conditions through education and to avoid aggravating factors, Morel focuses in the last chapter on the implementation of three practices that will lead, according to him, to the reformation and rebirth of the human species. The first concerns the application of the moral law, which should start with the family and end with the moralization of the whole. The second concerns the treatment by the "medical art" of the agents of intoxication. The third is addressed to society, which, aiming for public safety, should create mechanisms of defensive prevention through the marginalization of the deviant, and mechanisms of self-preservation through the modification of those conditions concerning the physical, psychic, and mental field, which are responsible for the differentiation of humans [2].

The reception of Morel’s theory

The idea that some characteristics of behavior and mental condition were mediated by heredity was not explicitly opposed by the early proponents of asylum reform and moral therapy. Esquirol identified "heredity" as a significant remote physical cause of insanity in the 1830s. By the middle of the 19th century, a closer look at family backgrounds appeared to have documented the extent to which asylum inmates had relatives who were likewise afflicted with mental illness or other social ills. Morel methodically molded these observations into a progressive degenerative framework [7].

The empirical and clinical observation of the medical history of psychiatric patients and their families together with the use of statistical data in Morel's treatises, were evidence of scientific gravity and validity for the physicians of the time about the importance of heredity. In the decades that followed, the histories of alcoholic families began to be examined in detail. It is not implausible that the concepts of degeneration and heredity were more widely accepted by psychiatrists in order to increase the significance of their specialty. The association of mental disorders with the brain created a biological framework. The inability to identify specific organic lesions as causes of the disorders gave room for heredity and degeneration to act as bridges that introduced these mental and neuropathological disorders into the field of general medicine [8]. Morel in his nosological classification also included "démence précoce", i.e., early dementia, which, later, would become the "dementia praecox" of German authors, including Emil Kraepelin [9].

His work formed the basis for the further development of psychiatry. The release of Charles Darwin's ''On the Origin of Species'', in 1859, helped expand the theory of degeneration through evolutionary theory and a more materialistic view of mental disorders; degeneration became synonymous with brain dysfunction and mental illness [10].

The references to degeneration and its association with social evolution passed through the medical-psychiatric view to many scientific disciplines with the theory being practically accepted by biology, anthropology, sociology, criminology, general psychiatry, and general pathology in the late 19th century and the first half of the 20th [11]. References to Morel's work can be found in many scientific texts of various countries of that time as there were many psychiatrists such as Cesare Lombroso, Henry Maudsley, Max Nordau, and others who embraced his views on the influence of the environment and the heredity of mental disorders and his biological explanation of criminality. The assumption that certain individuals or social classes were doomed to develop paranoia, commit criminal acts, or lead their generation to extinction because of their biological or physical heterogeneity, their physical inadequacy, or the particularity of their mental state, essentially shaped and influenced social policy in Europe in the second half of the 19th century [12].

The issue of the heredity of mental illnesses based on the theory of degeneration occupied psychiatrists from all over the world, but it also had as a consequence the emergence of another scientific view, "eugenics", which was formulated in 1883 by Francis Galton in ‘’Inquiries into Human Faculty and Its Development’’, which would shock the scientific world of the end of the 19th century as well as that of the 20th. Eugenics was supposed to study the socially controllable factors that could improve or reduce the racial qualities of future generations both physically and mentally. It would then become the ideology that would defend the intervention in human reproduction and its aim was to eliminate undesirable human groups, such as the mentally ill, with the aim to create the perfect human society. The incarcerated in the asylums were the easiest target for the application of eugenic measures, but not the only ones, because even the "apparently normal" people who were outside asylums and who proliferated unhindered, threatened the whole with erosion and decline [13].

After World War I, the eugenics theory was applied to the segregation and sterilization of "degenerates", culminating in Nazi Germany's "Final Solution" in World War II. As the theory of degeneration became politically brutal in the interwar years, most biologists moved away from it. After the war, the aversion to this theory became widespread [14]. In a sense, degeneracy was never disproved, just dismissed [15]. The effect of the theory on public opinion was significant and created new prejudices against those who deviated noticeably from the usual type of man or from the dominant aesthetic standards. The power of some of these prejudices remains to this day [16]. 

In modern times, genetics has accepted that all major mental disorders aggregate in family histories and are heritable. Research today aims to connect genetics with environmental factors, as well as with phenotypic characteristics, in order to gain a deeper understanding of the causes of mental disorders [17]. These factors are the ones Morel established in his theory as decisive for the study of psychiatric illness.

## Conclusions

Bénédict Augustin Morel's work was the first significant attempt to understand mental illness. Combining principles of biology, anthropology, medicine, and philosophy together with the belief in the existence of a primitive perfect human model, the French psychiatrist developed the theory of degeneration, with the aim of improving the physical, spiritual, and mental state of human societies and maintaining order in them. By creating a nosological framework, in which he mentioned the causes as well as the internal and external signs of mental disorders, he gave prestige and validity to the then-new branch of medicine, psychiatry. The belief that mental disorders can be inherited influenced social policy in the 19th and 20th centuries in many countries of the Western world. The impact of Morel's theory still survives today, as the factors he determined to be significant in understanding psychiatric disorders are the main focus of modern research and are considered key in interpreting the different causes and manifestations of mental illnesses.
